# Effects of an osteoporosis prevention training program on physical activity-related stages of change and self-efficacy among university students, Shiraz, Iran: a Randomized Clinical Trial

**Published:** 2014-10

**Authors:** MOHAMMAD HOSSIEN KAVEH, MONIRE GOLIJ, MAHIN NAZARI, ZOHREH MAZLOOM, ABBAS REZAEIAN ZADEH

**Affiliations:** 1 Department of Health Education and Promotion, Faculty of Health, Research Center for Health Sciences, Shiraz University of Medical Sciences, Shiraz, Iran;; 2 Department of Health Education and Promotion, Faculty of Health, Shiraz University of Medical Sciences, Shiraz, Iran;; 3 Department of Nutrition, Faculty of Nutrition and Science Food, Shiraz University of Medical Sciences, Shiraz, Iran;; 4 Department of Epidemiology, Faculty of Health, Shiraz University of Medical Sciences, Shiraz, Iran

**Keywords:** Empowerment, Osteoporosis, Behavior, Exercise, Self efficacy

## Abstract

**Introduction: **Osteoporosis is a major problem in today's world, being characterized by decreased bone mass and bone change. Due to deficiency of theory-based studies in young population, especially in students, there are significant knowledge gaps of effective planning. Thepresent study was performed in response to this need. The present study investigated the effect of an empowerment program on physical activity related stages of change and self-efficacyin preventing osteoporosis among university students.

**Methods:**In this randomized controlled trial (IRCT: IRCT201212016261N2), 152 female students of Shiraz University of Medical Sciences were selected through multi-stages cluster sampling and were randomly assigned to an experimental (n=76) and a control (n=76) group.The pre-and post-intervention data were collected using the Stages of Exercise Change Questionnaire (SECQ) of Marcos with Cronbach's alpha reliability of 0.89 and also the self-efficacy scale with a Cronbach's alpha reliability of 0.88 and Test-Retest Correlation Coefficient of 0.80. The educational intervention for the experimental group took place through problem-based learning method, small group discussion, and training manuals. In addition, training CDs and brochures were given to the subjects and short SMSs were sent to them. The data were analyzed throughSPSS, version 14, usingMann-Whitney test**,** Chi-square test, Wilcoxon and regression tests.

**Results:**Pre-intervention findings showed that participants had behavioral constructs below the expected levels. The results showed that the experimental group received significant statisticalincrease after the intervention in stage of change. Before the intervention, the mean scores of stages of changes in the experimental groups was 2.28±0.86 but this rose to 3±0.84 in the first post-test and 3.22±0.84 in the second post-test. The control group showed a significant increase in stage of change without intervention (pre-test 2.04±0.82, first post-test 2.18±0.87 and second post-test 2.3±0.89).However, this increase was more significant in the experimental group (p<0.001). The mean score of self-efficacy in physical activity in the intervention group upon completion of the course showed a significant increase (p<0.001). A significant correlation was found between the construct stage of change and self-efficacy.

**Conclusion:**Theory-based curriculum is effective in empowering individuals in stage of change and developing self-efficacy in physical activity of university students.

## Introduction

Osteoporosis is the most common non-communicable and metabolic disease (1, 2) that causes disability and diminished quality of life (3, 4). Women, as compared with men, are four times more likely to develop osteoporosis (5). In the Americas and Europe osteoporotic fractures account for 2.8 million disability-adjusted life years (DALYs) annually, and in Iran it is over 36761 years (6, 7). Although drug therapy may increase bone density and reduce fracture risk in postmenopausal women, the high cost and its side effects are also considerable. Evidence demonstrated the cost effectiveness of non-drug interventions based on lifestyle changes (8). According to the findings of some studies, one of the important elements in the prevention of osteoporosis is regular physical activity (9). But a large number of people in Iran and many countries are not getting recommended physical activity and are deprived of its benefits (10). Optimal effectiveness and sustainability of health promotion programs depend on application of such principles as target populations’ participation, and voluntary adoption and application of the changes (11). Empowerment is one of these principles (11, 12). It refers to increasing people’s control over decisions, lifestyle and activities that affect their health. This approach tries to develop self-esteem, self-management and self-control and make changes in behavior (12). Empowering has an important role in encouraging people to change their lifestyle (11). For education, the use of student-centered learning (SCL) such as problem-based learning and individual or group exploration for the purpose of empowerment is preferred (13).One of the principles and preconditions for the effectiveness of health promotion programs is the application of theory which helps to understand and determine the basic elements for behavior changes in the long-term (14).

Using theory is beneficial not only in needs assessment but also in development and evaluation based on the index of the intervention (14).

Training should be tailored to the characteristics and conditions of the target group. For behavior changes target group’s readiness is one of the important features. Different readiness for behavior change is shown in the Trans-theoretical Model (15). TTM model consists of stages of change and process of change. Stages of change classify individuals with different readiness levels for behavior changes in five phases including pre-contemplation, contemplation, preparation, action and maintenance. The next dimension of TTM model is the processes of change that shows the necessary elements for changing behavior and how to change the individual’s behavior in each stage (16).

The other structure found in the TTM model is self-efficacy which proposes that the feeling of ability to perform a specific task will affect behavioral choices and helps to deal with risk situation. Decisional balance is another structure in TTM model which focuses on advantages and disadvantages of behavior change (16).

Plotnikoff, et al.’s study demonstrated that the Trans-theoretical Model is able to predict the individual’s transition from stages of change (17). Sharifi-rad, et al. in a study showed that Trans-theoretical Model-based intervention caused a significant increase in self-efficacy scores and repelling skills against drug abuse in the experimental group as compared to that in the control group (18). Arneson and Ekberg’s study indicated that systematic empowerment of social support and group coherence among employees ought to be facilitated by the organization of a health-promotion arena (12).A review of the literature shows lack of interventional studies using the empowerment approach (1, 5, 8, 10), so the present study aimed to evaluate and determine the effect of an empowerment program on stages of change and self-efficacy in student's physical activity.

## Methods

This study was an educational randomized control trial at Shiraz University of Medical Sciences with Irct ID: IRCT201212016261N2. A total of 152 female students of Shiraz University of Medical Sciences were selected as the participants of this study, using a stratified- cluster sampling method. Our research (6345) was approved by Shiraz University Medical Sciences ethics committee and a signed informed consent was obtained from all participants. Shiraz University of Medical Sciences has 8 schools. Each of the schools of Shiraz university of Medical Sciences was considered as a subpopulation (stratum) and each of the training courses as a cluster. Three out of 8 schools, Pharmacy, Dental and Medical schools were selected because they had an under-graduate field.Also we aimed to study the control and experimental groups, which were in the same class, in each college. 

So, eight schools were re-categorized in six clusters. Among the six schools, four including Public Health, Nursing & Midwifery, Rehabilitation Sciences and Management &Medical Informatics were selected through simple random sampling. Then, 2 disciplines of study that had students in under-graduate levels at the time of this study were randomly selected from every college. Finally, the selected two groups in each school were randomly assigned to the experimental (n=76) and control (n=76) groups.

The experimental group underwent an educational intervention forfour 120-minute sessions, using interactive teaching-learning methods such as problem-based learning (PBL) and group discussion in addition to video clips. Learner-based methods involve a 7-step learning model based on the Maastricht problem (19, 20).

In addition, training CDs and brochures were given to the subjects and short SMSs were sent to them from cell phone in order to repeat the points taught in the last session and increase the participants’ learning. 

Training topics included rethinking our health, recognizing threats, dimensions of osteoporosis, ways of preventing osteoporosis, intention, planning and implementing. 

One week after the end of the educational intervention, the first post-test was given to the experimental and control groups to evaluate the changes made through educational intervention, and six weeks after the educational intervention, the post-test was repeatedto evaluate the durability of the learned information by participants in both groups.

Progress in self-efficacy and stage of change during the two stages (one week and six weeks after training), was used as the criteria for successful intervention. 

We used SECQ questionnaire (Stages of Exercise Change Questionnaire) developed by Marcus and colleagues (1992) to measure stages of change in physical activity. The described stages in terms of exercise behavior include: 1-pre-contemplation (no exercise and no intention to start it in six months) 2- contemplation (no exercise but want to start it in the next six months) 3- preparation (has decided to do exercise at a standard level in the next month) 4- act (in the six past months started to do exercise at a standard level) and 5- maintenance(is exercising more than six months at the standard level). The standard level in doing exercise is defined as three times a week, each time lasting 20 minutes. Kappa coefficient for the validity of the questionnaire has been reported 78by Karimzadeh and colleagues (21).

To measure self-efficacy in physical activity, we created a questionnaire by using the existing questionnaires, authentic literature (21, 22) and also experts’ ideas. This questionnaire consisted of9 items with 5 point Likert response scale. Its content validity was examined by 6 health professionals. In order to determine the face validity in addition to receiving experts’ ideas, the questionnaire was administered in a sample of students and necessary corrections were made based on the feedback received.

**Statistical analysis**

The data were analyzed using SPSS software, version 14. Chi-square test, Friedman, Wilcoxon and Kruskal-Wallis tests were used to compare changes in the mean score of stage of change score and self-efficacy scores for the experimental and control groups during the pre-test phase (pre-intervention) and post-tests (oneweek and six weeks after intervention). Also Pearson correlation coefficient was used to check the relationship between stages of change score and self-efficacy score in the experimental and control groups during the pre-test and post-tests phases (one-week and six weeks after the intervention). 

## Results

The results showed that there were no statistically significant differences in age, college of education, Body Mass Index, education level and occupation of parents between the two groups. The Students’ mean age in the experimental group was 20.20±1.395 and in the control group was 20.08±0.813. The mean Body Mass Index in the experimental group was 21.23±2.642 and in the control group it was 20.83±2.775. 

23% of the subjects in the intervention group and 19% of the control group were majoring in the public health and nutrition college; 18% of the experimental group and 16% of the control group were in thenursing and midwifery college. Also 20% of the intervention group and 26% of control group were in the management and medical information college; 15% of the experimental group and 15% of the controls were enrolled in the rehabilitation college.21% of the students in the experimental group and 19.7% of those in the control group stated that there was one person with osteoporosis in their family. 

As can be seen in Table 1, in the experimental group before the intervention, 9 students(11.8%) were in the pre-contemplation stage, 47 (61. 8%) in the contemplation stage, 11 (14.5%) in the preparation stage, 8 (10.5%) in the act stage, and 1 (1.3%) in the maintenance phase. Distribution of this group in terms of stages of change one week after the intervention was: 1 student(1.3%) in the pre-contemplation stage, 22 (28.9%) in the contemplation stage, 31(40.8%) in the preparation stage, 20 (26.3%) in the action phase, and 2 (2.6%) in the maintenance phase.

Six weeks after the implementation of such a curriculum, the frequency distribution of the experimental group included 1 student(1.3%) in the pre-contemplation stage, 13 (17.1%) in the contemplation stage, 25 (32.9%) in the preparation, 35 (46.1%) in the act, and 2 (2.6%) in the maintenance phase. There is a progressive change in the control group as can be seen; however, more progress is observed in the experimental group. 

**Table 1:** Descriptive findings on physical activity-related stages of change among the groups in three phases of measurement

**Groups** **Stages of ** **change**	**Pre-intervention**		**One week after intervention**		**6 weeks after intervention**	
	**Experimental**	**Control**	**Experimental**	**Control**	**Experimental**	**Control**
	**Frequency (%)**	**Frequency (%)**	**Frequency (%)**	**Frequency (%)**	**Frequency (%)**	**Frequency (%)**
**Pre-contemplation**	9 (%11.8)	16 (%21.1)	1 (%1.3)	13 (%17.1)	1 (%1.3)	9 (%11.8)
**Contemplation**	47 (%61.8)	48 (%63.2)	22 (%28.9)	45 (%59.2)	13 (%17.1)	46 (%60.5)
**Preparation**	11 (%14.5)	6 (%7.9)	31 (%40.8)	10 (%13.2)	25 (%32.9)	12 (%15.8)
**Action**	8 (10.5)	5 (%6.6)	20 (%26.3)	7 (%9.2)	35 (%46.1)	7 (%9.2)
**Maintenance **	1 (%1.3)	1 (%1.3)	2 (%2.6)	1 (%1.3)	2 (%2.6)	2 (%2.6)

Our results also showed that there was no significant difference in the mean scores of the stages of change in physical activity before educational intervention (z=-0.943, p=0.052), while a week after the educational program (z=-5.604, p<0.001) and six weeks after the intervention (z=-6.486, p<0.001), a statistically significant difference was found between the two groups (Table 2).

**Table 2:** Comparison of the mean scores of stages of change between the groups through phases of measurement

**Groups** **Stages of change**	**Experimental**	**Control**	**Mann-Whitney test**
**Pre-intervention (Mean±SD)**	2.28±0.86	2.04±0.82	p=0.052
**One week after intervention (Mean±SD)**	3±0.84	2.18±0.87	p<0.001
**6 weeks after intervention (Mean±SD)**	3.32±0.84	2.3±0.89	p<0.001
**Friedman test**	p<0.001	p<0.001	

The internal consistency of self-efficacy was approved through the pilot study on 41 students and Cronbach's alpha reliability was calculated to be 0.88. In addition, test-retest reliability of this instrument (r=0.80, p<0.001) was also desirable. The mean scores of self-efficacy at three measuring phases (pre-intervention, one week and six weeks after training), using Friedman test, showed that during the intervention the training group had significant improvements (X^ 2 ^=24.713, df=2, p<0.001), but the results of these tests showed no significant improvement in the control group. (X^ 2 ^=5.567, df =2, p=0.062).

Self-efficacy mean scores in physical activity between the two groups in each measuring phase showed that before the intervention, groups had no significant difference in terms of self-efficacy (z=-0.459, p=0.646), but one week after the intervention (z=-2.750, p=0.006) and six weeks after it (z=-3.690, p<0.001), a statistically significant difference in self-efficacy scores in physical activity was seen in both groups (Table3). 

The results of the correlation test showed a positive correlation between the stages of change and self-efficacy in physical activity before the intervention (r=0.740, p<0.001), one week (r=0.437, p<0.001) and six weeks (r=0.314, p=0.006) after the intervention. 

**Table 3:** Comparison of the mean scores of self-efficacy between the groups through phases of measurement

**Self-efficacy** **Groups**	**Pre-intervention**(Mean±SD)	**One week after intervention**(Mean±SD)	**6 weeks after intervention**(Mean±SD)	**p **
**Experimental**	22.79±7.38	26.68±7.18	27.54±7.36	p<0.001
**Control**	21.87±7.71	23.29±7.56	22.55±7.58	p=0.062
**Mann-Whitney test**	p=0.646	p=0.006	p<0.001	

## Discussion

The importance of theory-based interventions in prevention of osteoporosis has been shown in several studies (1, 21, 23). The findings of the present study confirmed the effectiveness of educational intervention in improving the variables.

We did not find similar studies in the field of youth empowerment in stages of change and self-efficacy in physical activity to osteoporosis prevention.

Considering the readiness of the individuals to change behavior during the training sessions is an important principle. The studies by Karimzadeh (2007), Annesi (2010), and Moeini (2010) have confirmed this principle (21, 24, 25). Readiness of individuals for changing behavior is described by stage of change structure in TTM model (15).

In our study, most participants were in the pre-action stage(88.2% in the intervention group and 91.1% in the control group).Both the control and experimental groups showed improvement in stages of change after the educational program; however, the change was more significant in the experimental group. Increase in the number of participants in the experimental group in preparation and action stagesafter the educational intervention can be related to the education given to them. 

Annesi (2010), Zhang and (2006) and Hoke (2011) in their studies emphasized the relationship between training intervention and improvement in stages of change (24, 26, 27). Karimizadeh and Moeini showed that training was able to facilitate the transition of people in stage of change (21, 25).

In our study, it was assumed that the educational intervention, based on the TTM model, could improve the individual’s self-efficacy in physical activity. Based on our results, the experimental group showed a significant improvement in self-efficacy in physical activity as compared to that in the control group during the intervention. 

Regarding self-efficacy, before the intervention, there was no significant difference between the two groups, but after the intervention the experimental group showed a statistically significant difference.

Sharifirad’s report showed that the educational intervention based on Trans-theoretical Model made significant increases in the self-efficacy and coping skills against substance abuse in the experimental group as compared with the control group (18). Also the effectiveness of training on self-efficacy improvement has been demonstrated in other studies (24, 28, 29).

According to our results, there are significant positive correlations between the stages of change and self-efficacy in physical activity.Based on the relationship between stages of change and self-efficacy, the cause and effect relationship cannot be represented. This indicates that increased self- efficacy can lead to the stages of change improvement. Gaining experience can lead to increased self-efficacy. On the whole, there should be an attempt in the educational intervention to improve the stages of change and increase self-efficacy, or both. 

Some other studies reported a significant correlation between improvement in the stages of change and increase in self-efficacy (24, 30, 31).

In this research, for implementing educational intervention interactive methods such as problem-based learning (PBL) and small group discussion technique,as well as providing opportunities for questions and answers during and after training sessions, were used.

Evidence shows that interactive teaching methods such as PBL are a more effective to achieve competencies of public health than traditional teaching methods.In loureiros’ study (2008), it has been demonstrated that problem-based learning approach (PBL) is an appropriate procedure for planning in the public health field and the acquisition of basic public healthcompetence (32).

Some studies support the effectiveness of this method compared with other training methods. Fesharakis’ study (2010) confirmed that learning in PBL method causes deeper understanding of the contents because of spending more time in the study by the learner (33).

Arneson and Ekberg (2005) emphasized that the use of interactive teaching approaches, such as problem-based learning leads to empowerment of the people, thereby increasing their knowledge and also self-efficacy. This has led to an increase in the willingness of individuals and participants to move forward in the stages of change, examine their working conditions and determine the problems and find solutions (12).Overall, the findings of this study indicate the effectiveness of the used educational intervention in the improvement of physical activity and self-efficacy.

## Conclusion

According to the results of the present study, developing and implementing educational interventions based on using interactive teaching methods and appropriate patterns should be considered for adolescents and young adults. Also, further trials using various methods and theories are recommended.

**Limitations& Suggestions**

In this study although measuring physical activity and self-efficacy, according to the participants’ reports, is a common practice, the bias in reporting is likely. In this study, although in the process of sampling, colleges were randomly selected, the results may not be applicable to other schools. This study had no long-term follow-up, and judgment cannot be made about the effects of long-term intervention and we cannot predict continuity of the results in the longterm. In addition, the results may not be in compliance with those obtained through interventions based on other educational modalities such as e-learning or a combination of education and counseling interventions.

It is recommended to design and implement educational trials to change and improve teachers and parents’ lifestyle, thereby changing students' lifestyle to prevent osteoporosis. Also long-term effects of such educational programs should be assessed.

**Ethical consideration**

The educational administrators gave the authorization for the study before research implementation. Initially, the participants signed a testimonialwhich included an introduction to the study, the objectives and implementation method. In addition, participants were assured that the information in thequestionnaires remain secret. Meanwhile, participation in the study was voluntary. After the research implementation, some of educational contents which were main points were offered to the control group for ethical consideration.

**Implication**

The findings of this study can be used wherever prevention of osteoporosis is taught including schools, universities, etc. Teachers can provide appropriate opportunities to empower learners by designing effective teaching strategies. Educational planners and administrators should emphasize the use of specific strategies for empowerment. It is recommended that in the behavioral research the change behavioral model should be used and increase of self-efficacy should be focused. 
